# Practical Observations, Showing That Mercury Is the Sole Cause of What Are Termed Secondary Symptoms

**Published:** 1840-01

**Authors:** 


Art. I.—*Practical Observations, showing that Mercury is the Sole Cause of what are termed Secondary Symptoms.* By P. J. Murphy, m.d.—*London,* 1839. 8vo, pp. 107.

The substance, or rather the shadow of Dr. Murphy's book, is the following doctrine : "That ulceration of the throat, cutaneous eruptions, iritis, nodes, venereal swelled testis, &c., and termed secondary symptoms, are produced solely by mercury." (p. 103.) The title-page sets forth the same notion, but the book itself, somewhat strangely, contains no proofs of its truth, unless the author's earnest conviction and some loose reasoning, can be called proofs. Dr. Murphy seems to have been unconscious that the truth of such a theory can only be established by facts, and ignorant that a large number of facts utterly contradict it. Thus, within the last few years, 80,000 cases of primary ulcerations of the genitals have been submitted to anti-mercurial treatment in France, Sweden, and Hamburg, "and the proportion of secondary affections, where the primary symptoms have been treated without mercury, is, at its lowest estimate, reduced to ten, at its highest to twenty, in the hundred." (*British and Foreign Medical Review*, Vol. V., p. 7.) In this country, we thought everybody knew that similar experiments were made in our own army, twenty years ago. From the circular, published by the Army Board, dated 2d April, 1819, it appears that in 1940 cases of primary ulceration of the genitals, occurring between Dec. 1816 and Dec. 1818, treated without mercury, 96 cases of secondary symptoms occurred : during the same period, 2827 cases of ulceration of the genitals were treated with mercury, out of which but 51 had secondary symptoms. Much more evidence of a similar kind could be adduced; but this (which is of the strongest kind, affirmative, not negative), sufficiently proves, that secondary symptoms do occur, when no mercury whatever has been given; and thus Dr. Murphy's notion, that secondary symptoms are produced solely by mercury, is totally disproved. We would suggest to Dr. M., before he theorizes and writes again, to make himself well acquainted with the history of the disease he undertakes to elucidate, and with the opinions and practice of others. This he has not done in the present instance. We have no inclination to criticise more minutely his book, lest we should ourselves run the risk of being classed with those modern critics, who have been described as "butchering the defenceless rabble of the lame and halt, who venture with such courage, in our days, into the literary tilt-yard."

The propriety of the non-mercurial treatment, which Dr. M. recommends, does not depend on the truth of his hasty generalization. The absolute *necessity* for mercury, in the majority of cases, has been disproved ; the question still remains open as to its *expediency*.

